# Marine Litter Distribution and Density in European Seas, from the Shelves to Deep Basins

**DOI:** 10.1371/journal.pone.0095839

**Published:** 2014-04-30

**Authors:** Christopher K. Pham, Eva Ramirez-Llodra, Claudia H. S. Alt, Teresa Amaro, Melanie Bergmann, Miquel Canals, Joan B. Company, Jaime Davies, Gerard Duineveld, François Galgani, Kerry L. Howell, Veerle A. I. Huvenne, Eduardo Isidro, Daniel O. B. Jones, Galderic Lastras, Telmo Morato, José Nuno Gomes-Pereira, Autun Purser, Heather Stewart, Inês Tojeira, Xavier Tubau, David Van Rooij, Paul A. Tyler

**Affiliations:** 1 Center of the Institute of Marine Research (IMAR) and Department of Oceanography and Fisheries, University of the Azores, Horta, Portugal; 2 Laboratory of Robotics and Systems in Engineering and Science (LARSyS), Lisbon, Portugal; 3 Institut de Ciències del Mar (ICM-CSIC), Barcelona, Spain; 4 Norwegian Institute for Water Research (NIVA), Marine Biology section, Oslo, Norway; 5 Ocean and Earth Science, University of Southampton, National Oceanography Centre, Southampton, United Kingdom; 6 Norwegian Institute for Water Research, Bergen, Norway; 7 Alfred-Wegener-Institut, Helmholtz-Zentrum für Polar- und Meeresforschung, Bremerhaven, Germany; 8 GRC Geociències Marines, Departament d′Estratigrafia, Paleontologia i Geociències Marines, Facultat de Geologia, Universitat de Barcelona, Campus de Pedralbes, Barcelona, Spain; 9 Marine Biology & Ecology Research Centre, Marine Institute, Plymouth University, Plymouth, United Kingdom; 10 Netherlands Institute for Sea Research (NIOZ), Texel, The Netherlands; 11 Institut Français de Recherche pour l′Exploitation de la Mer (IFREMER), Bastia, France; 12 National Oceanography Centre, University of Southampton Waterfront Campus, Southampton, United Kingdom; 13 OceanLab, Jacobs University Bremen, Bremen, Germany; 14 British Geological Survey, Murchison House, Edinburgh, United Kingdom; 15 Portuguese Task Group for the Extension of the Continental Shelf (EMEPC), Paço de Arcos, Portugal; 16 Renard Centre of Marine Geology (RCMG), Department of Geology and Soil Science, Ghent University, Gent, Belgium; Bangor University, United Kingdom

## Abstract

Anthropogenic litter is present in all marine habitats, from beaches to the most remote points in the oceans. On the seafloor, marine litter, particularly plastic, can accumulate in high densities with deleterious consequences for its inhabitants. Yet, because of the high cost involved with sampling the seafloor, no large-scale assessment of distribution patterns was available to date. Here, we present data on litter distribution and density collected during 588 video and trawl surveys across 32 sites in European waters. We found litter to be present in the deepest areas and at locations as remote from land as the Charlie-Gibbs Fracture Zone across the Mid-Atlantic Ridge. The highest litter density occurs in submarine canyons, whilst the lowest density can be found on continental shelves and on ocean ridges. Plastic was the most prevalent litter item found on the seafloor. Litter from fishing activities (derelict fishing lines and nets) was particularly common on seamounts, banks, mounds and ocean ridges. Our results highlight the extent of the problem and the need for action to prevent increasing accumulation of litter in marine environments.

## Introduction

Litter disposal and accumulation in the marine environment is one of the fastest growing threats for the world's oceans health. Marine litter is defined as ‘‘any persistent, manufactured or processed solid material discarded, disposed of or abandoned in the marine and coastal environment”[1]. The issue has been highlighted by the United Nations Environment Program [1] and was included in the 11 Descriptors set by Europe's Marine Strategy Framework directive (2008/56/EC) (MSFD) [2]. The MSFD requires each Descriptor in all European marine waters not to deviate from the undisturbed state and reach Good Environmental Status (GES) by 2020.

With an estimated 6.4 million tonnes of litter entering the oceans each year [1], the adverse impacts of litter on the marine environment are not negligible. Besides the unquestionable aesthetic issue, litter can be mistaken for food items and be ingested by a wide variety of marine organisms [3]–[8]. Entanglement in derelict fishing gear is also a serious threat, particularly for mammals [9]–[11], turtles [12] and birds [13] but also for benthic biota such as corals [14], [15]. High mortality of fish through “ghost fishing” is another consequence of derelict fishing gear in the marine environment [16]. Moreover, floating litter facilitates the transfer of non-native marine species (e.g. bryozoans, barnacles) to new habitats [17], [18]. Barnes et al. [19] estimated that the dispersal of alien species through marine litter more than doubles the rate of natural dispersal processes, especially during an era of global change.

Although the type of litter found in the world's oceans is highly diverse, plastics are by far the most abundant material recorded [20]–[22]. Because of their persistence and hydrophobic nature, their impact on marine ecosystems is of great concern. Plastics are a source of toxic chemicals such as polychlorinated biphenyls (PCBs) and dioxins that can be lethal to marine fauna [23]. Furthermore, the degradation of plastics generates microplastics which, when ingested by organisms, can deliver contaminants across trophic levels [24]–[27].

Litter type, composition and density vary greatly among locations and litter has been found in all marine habitats, from surface water convergence in the pelagic realm (fronts) down to the deep sea where litter degradation is a much slower process [21]. The spatial distribution and accumulation of litter in the ocean is influenced by hydrography, geomorphological factors [21], [28], prevailing winds and anthropogenic activities [29]. Hotspots of litter accumulation include shores close to populated areas, particularly beaches [30], but also submarine canyons, where litter originating from land accumulates in large quantities [28], [31].

In Europe, much has been written on the abundance and distribution of litter on the coastline and in surface waters [32]–[41]. As more areas of Europe's seafloor are being explored, benthic litter is progressively being revealed to be more widespread than previously assumed [15], [28], [29], [31], [42]–[52]. The sources of litter accumulating on the seafloor are variable, depending upon interactions between distances from shore [31], [45], oceanographic and hydrographic processes [47] and human activities such as commercial shipping [29] and leisure craft [43].

Early studies used trawling to quantify litter abundance on the seafloor [53], whilst more recent studies have demonstrated the potential of remotely operated vehicles (ROV), manned submersibles or towed cameras to study litter in the deep sea [15], [31], [43], [47], [54], [55]. However, understanding spatial patterns in litter abundance and distribution in the deep sea is challenging, owing to the lack of standardization in the sampling and analytical methodologies used. Furthermore, the high cost of sampling in the deep sea has limited our ability to perform standardized surveys across large areas to understand fully the extent of this pollution issue.

The problem of marine litter on the deep seafloor was addressed by the EU-FP7 project HERMIONE, recognising the need to use the surveys conducted by all partners (although designed for other purposes) to gather data on litter in the deep sea. This paper presents the results on the distribution and densities of marine litter obtained during these surveys, with additional data provided by the UK's Mapping the Deep project as well as other previous projects. It provides a unique large-scale analysis of litter on the seafloor across different physiographic settings and depths.

## Materials and Methods

### Study areas

**Figure 1:**
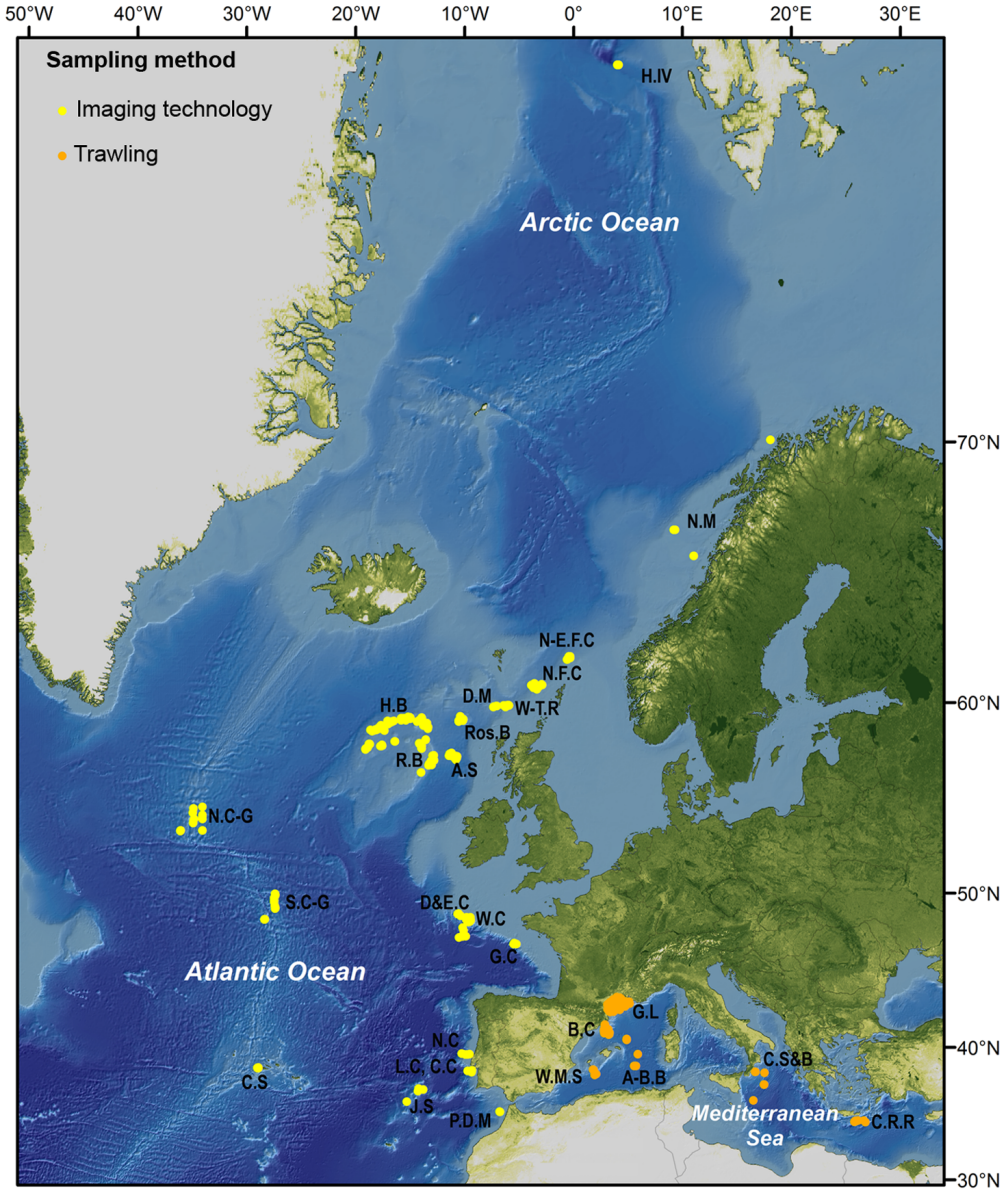
Locations of the study sites sampled with imaging technology (ROVs, manned submersible, towed camera systems) and trawling. A-B.B  =  Algero-Balearic Basin (W. Med.), A.S  =  Anton Dohrn Seamount, B.C  =  Blanes Canyon (NW Med.), C.C  =  Cascais Canyon, C.S  =  Condor Seamount, Calabrian Slope & Basin  =  C.S&B, Crete-Rhodes Ridge  =  C.R.R, D&E.C  =  Dangeard & Explorer Canyons, D.M  =  Darwin Mounds, G.L.C  =  Gulf of Lion canyons (NW Med.), G.L  =  Gulf of Lion, G.C  =  Guilvinec Canyon, H.B  =  Hatton Bank, H.IV  =  HAUSGARTEN, station IV, J.S =  Josephine Seamount, L.C =  Lisbon Canyon, N.C  =  Nazaré Canyon, N.C-G  =  North Charlie Gibbs Fracture Zone, N-E.F.C  =  North-East Faroe-Shetland Channel, N.F.C  =  North Faroe-Shetland Channel, N.W =  Norwegian margin, P.D.M  =  Pen Duick Alpha/Beta Mound, R.B  =  Rockall Bank, Ros.B  =  Rosemary Bank, S.C  =  Setúbal Canyon, S.C-G  =  South Charlie Gibbs Fracture Zone, W.C  =  Whittard Canyon, W.M.S  =  Western Mediterranean slope, W-T.R  =  Wyville-Thomson Ridge.

Data were gathered from surveys conducted during research cruises led by various European institutions between 1999 and 2011. A total of 32 sites in the northeastern Atlantic Ocean, Arctic Ocean and Mediterranean Sea were surveyed (Table 1; Figure 1). Surveyed sites were located on continental shelves and slopes, submarine canyons, seamounts, banks, mounds, ocean ridges and deep basins, at depths ranging from 35 to 4500 meters (Table 1).

**Table 1:** Sampling locations, date and methods for the collection of data on litter along with litter densities (mean number of items ha^−1^ and kg ha^−1^ ± standard errors).

Location	Year	Method	N° of samples	Mean depth (m)	Density (n ha^−1^)	Density (kg ha^−1^)	Area covered (ha)
**ATLANTIC**							
***Continental slopes***							
North Faroe-Shetland Channel	2006	TC	19	657	0.3±0.2	-	2.3
North-East Faroe-Shetland Channel	2006	TC	11	501	1.9±1.0	-	1.2
***Continental shelf***							
Norwegian Margin	2007	SUB	9	304	9.7±3.8	-	0.6
***Submarine canyons***							
Dangeard & Explorer Canyons	2007	TC	44	578	7.2±2.7	-	3
Nazaré Canyon	2007	ROV	13	3144	4.2±1.6	-	9
Lisbon Canyon	2007	ROV	1	1602	66.2	-	1
Setúbal Canyon	2007	ROV	1	2194	24.6	-	0.9
Cascais Canyon	2007	ROV	1	4574	10.6	-	1
Guilvinec Canyon	2008–2010	ROV	8	661	31.9±28.1	-	4.1
Whittard Canyon	2010	ROV-TC	11	2668	1.4±0.4	-	12.4
***Seamounts, banks and mounds***							
Anton Dohrn Seamount	2005–2009	TC	24	992	1.9±1.0	-	2.2
Condor Seamount	2010–2011	ROV	48	258	14.6±3.0	-	5.6
Josephine Seamount	2012	ROV	4	1455	5.7±3.3	-	0.9
Hatton Bank	2005–2011	ROV-TC	52	706	1.9±0.8	-	4
Rockall Bank	2005–2011	ROV-TC	29	702	0.7±0.5	-	2.4
Rosemary Bank	2006	TC	14	577	3.3±2.3	-	1.1
Pen Duick Alpha/Beta Mound	2009	ROV	7	534	2.5±1.7	-	1.1
Darwin Mounds	2011	ROV	7	1007	9.7±2.9	-	1.8
***Ocean ridges***							
North Charlie Gibbs Fracture Zone	-	ROV	24	2300	0.4±0.3	-	2.4
South Charlie Gibbs Fracture Zone	-	ROV	24	2600	2.9±1.4	-	2.4
Wyville-Thomson Ridge	2006	TC	15	670	10.9±4.3	-	1.2
**MEDITERANEAN**							
***Continental slopes***							
Calabrian Slope (Central Med.)	2009	Trawl	4	1400	-	0.6±0.4	18.9
Western Mediterranean Slope	2009	Trawl	8	1500	-	4±1.8	56
Crete-Rhodes Ridge (E. Med.)	2009	Trawl	8	1500	-	1.1±0.3	37.9
Blanes slope (NW Med.)	2009	Trawl	94	1387	-	1.2±0.4	407
***Continental shelf***							
Gulf of Lion (NW Med.)	2009	Trawl	52	85	0.4±0.1	-	276.4
***Submarine canyons***							
Blanes Canyon (NW Med.)	2009–2011*	ROV-Trawl	4 (13)	1496(1431)	32.1±11.9	0.7±0.2	2(33.9)
Gulf of Lion Canyons (NW Med.)	2009	Trawl	11	510	0.4±0.1	-	126.5
***Deep basins***							
Algero-Balearic Basin (W. Med.)	2009	Trawl	3	2883	-	1.8±1.5	16
Crete-Rhodes Ridge (E. Med.)	2009	Trawl	2	3000	-	1.2±0.3	2.8
Calabrian Basin (Central Med.)	2009	Trawl	3	2967	-	1.7±0.6	12.5
**ARCTIC**							
***Continental slope***							
HAUSGARTEN, station IV	1999–2011	TC-ROV	10	2450	13.6±7.9	-	72.2

*Numbers in parentheses refer to trawl surveys. ROV =  remotely operated vehicle; TC  =  towed camera system; TRAWL  =  Otter Trawl or Maireta System; SUB  =  manned submersible.

### Sampling methods

Sampling methods included both imaging technology (still photograph and video) and fishing trawls (Figure 1; Table 2). The Atlantic sites were surveyed uniquely using imaging technology, whilst sites located in the Mediterranean Sea were primarily investigated by trawling (except for some ROV transects in the Blanes submarine canyon). Video footage was collected by different ROVs (*Genesis*, *Isis*, *Liropus*, *Luso*, *Lynx*, *SP* and *Victor 6000*), manned submersible (*JAGO*, GEOMAR) and towed camera systems (Seatronics and the HD-video hopper video system). Still photographs were taken with the Ocean Floor Observation System (OFOS) at the HAUSGARTEN observatory, station IV. Technical details about each platform can be found elsewhere (see Table 2). Trawl samples were collected using two different gears: a net (GOC 73) with a 20 mm-diamond stretched mesh size at the cod-end [56] and an otter trawl Maireta System (OTMS), with a cod-end mesh size of 40 mm and an outer cover of 12 mm [29], [57].

**Table 2:** Information on each platform used to collect video and photographs for the collection of data on litter densities and distribution on the seafloor of European waters.

Sampling platform	Name	Format	N° of samples	Total area surveyed (m^2^)	Field of view (m)	References
**Manned submersible**	*Jago*	video	13	5561	1.5	[95]
**ROVs**	*Luso*	video	8	35587	3.6–4.4	[15]
	*Sp*	video	44	29749	2.3	[15]
	*Isis*	video	64	167308	2.0	[31]
	*Genesis*	video	20	86700	2.6	[96]
	*Liropus*	video	4	19867	3.0	[97]
	*Lynx*	video	19	3750	1.0	[98]
	*Victor 6000*	video	6	421840	10.0	[46]
**Towed camera systems**	*Seatronics*	video	194	158528	1.5	[99]
	*HD video hopper system*	video	6	21490	3.0	[100]
	*Ocean Floor Observation System*	photographs	2882	8570	0.8–11.6	[43]

Further technical information about each platform can be found in the indicated references.

### Analysis of image data

Protocols for video analysis varied slightly according to the platform used, but followed the same general outline. The entire footage was visualised and the number of litter items and depth recorded. Each litter item was classified into six different categories: plastic (all plastic with exception of fishing line and net), derelict fishing gear (fishing line or net), metal, glass, clinker (residue of burnt coal). Because of the low densities found at all sites, paper and cardboard, fabric, wood and unidentified items were grouped in the same category (other items). Although fishing lines and nets are mostly made of plastic, fishing gear was considered as a separate litter category because of our knowledge on its source and social implications and the particular impacts of this type of litter, such as ghost fishing and entanglement.

For each dive (sample), the area covered was calculated by multiplying the linear distance on the seafloor (off bottom footage were excluded from the analysis) by the average width of view of each of the platforms (Table 2).

For data derived from still photographs (OFOS), all images along each transect (taken at 30 s to 50 s-intervals) were analysed for the presence of litter items. Parallel laser points on the images allowed calculations of the area for each image; ranging between 0.8 and 11.6 m^2^. For OFOS, each image was considered to be a separate sample, while for video data, each dive was considered a single sample.

### Trawl data

Hauls in the Gulf of Lion (shelf and submarine canyons) were performed with a bottom trawl equipped with a GOC 73 net [56]. After trawling, litter items were counted and classified into the different categories (see above).

Trawling at the other Mediterranean sites was performed using an otter trawl Mareita System (OTMS). All litter items were separated and classified into different categories (see above) and weighed, after excess water and mud had been removed. The use of weight rather than number to quantify litter was based on the high abundance of broken plastics (from whole plastic bags to very small (<0.5 cm) pieces of plastics) and broken glass, which impeded the quantification of single items without overestimating abundances of certain categories over others [29].

### Data analysis

For each sample (video and still photographs), litter density was estimated as items of litter hectare^−1^ (ha; 10,000 m^2^) of seafloor surveyed. For trawl data where litter was measured in weight, litter density was estimated as kg of litter ha^−1^. Sites were grouped into 6 different groups according to physiographic characteristics (Table 1); (1) continental shelves; (2) continental slopes (excluding submarine canyons); (3) submarine canyons; (4) seamounts, banks and mounds; (5) ocean ridges and (6) deep basins. Tests for investigating differences among litter densities across physiographic settings were done separately according to the unit in which litter density was estimated (number ha^−1^ or weight ha^−1^). For both cases, the data were not normally distributed but variances were equal, therefore, the non-parametric Kruskal-Wallis rank sum test followed by a multiple comparison test (Dunn's pairwise comparison) were performed using the statistical package R. Variation in litter composition between physiographic settings were tested for significance using ANOSIM (Analysis of similarity) in PRIMER v6 software [58]. Bray-Curtis similarity [59] was calculated on log(x+1) transformation of the percentage contribution of litter type for each of the physiographic settings, across the entire data set. A similarity percentage analysis (SIMPER) was applied to identify the discriminating feature of the dissimilarities and similarities between physiographic settings.

## Results

### Litter density

**Figure 2:**
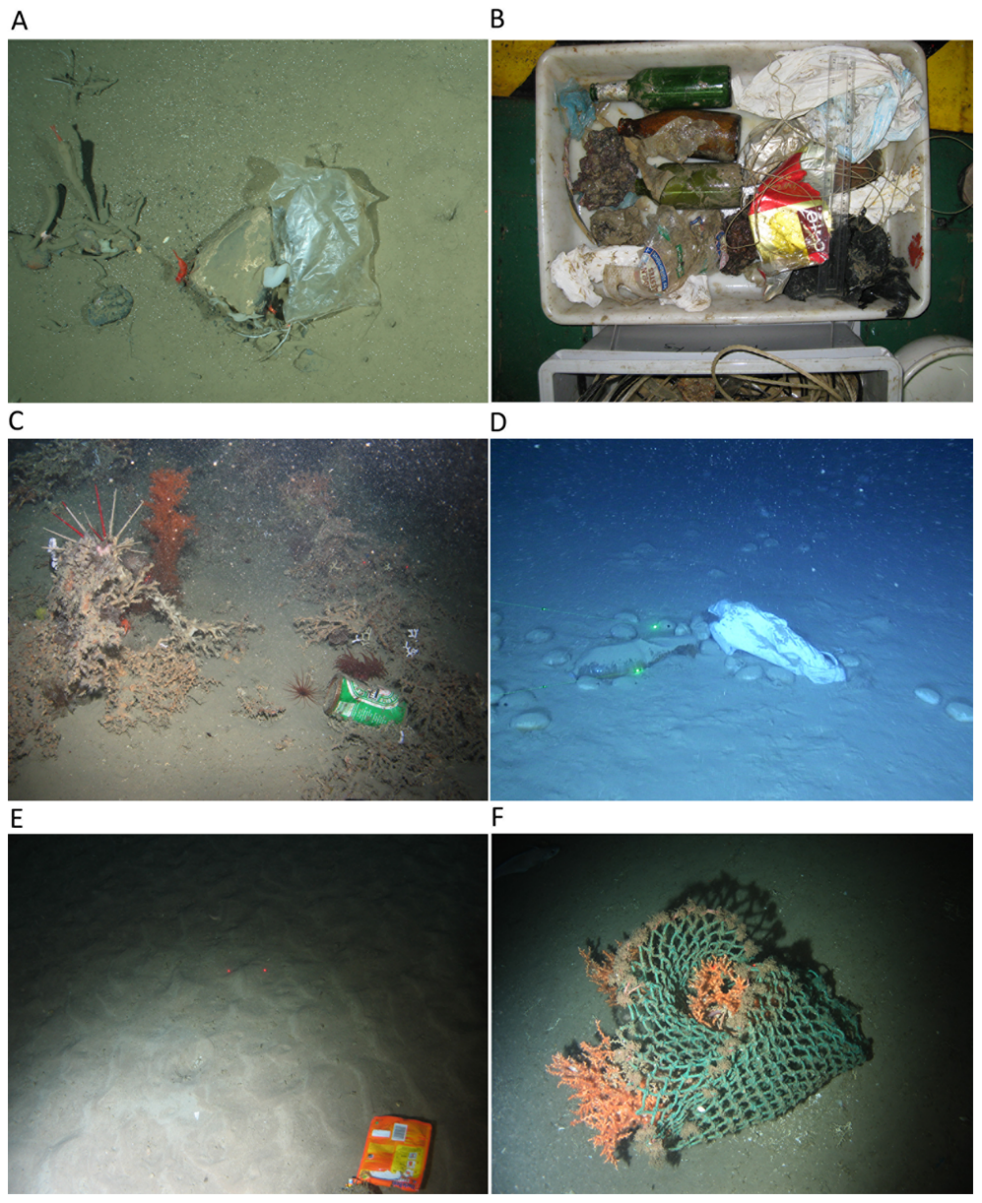
Litter items on the seafloor of European waters. A  =  Plastic bag entrapped by a small drop stone harbouring sponges (*Cladorhiza gelida*, *Caulophacus arcticus*), shrimps (*Bythocaris* sp.) and a crinoid (*Bathycrinus carpenterii*) recorded by an OFOS at the HAUSGARTEN observatory (Arctic) at 2500 m; B  =  Litter recovered within the net of a trawl in Blanes open slope at 1500 m during the PROMETO V cruise on board the R/V “García del Cid”; C  =  “Heineken” beer can in the upper Whittard canyon at 950 m water depth with the ROV Genesis; D  =  Plastic bag in Blanes Canyon at 896 m with the ROV “Liropus”; E  =  “Uncle Benn's Express Rice” packet at 967 m in Darwin Mound with the ROV “Lynx” (National Oceanography Centre, UK); F  =  Cargo net entangled in a cold-water coral colony at 950 m in Darwin Mound with the ROV “Lynx” (National Oceanography Centre, UK).

**Figure 3:**
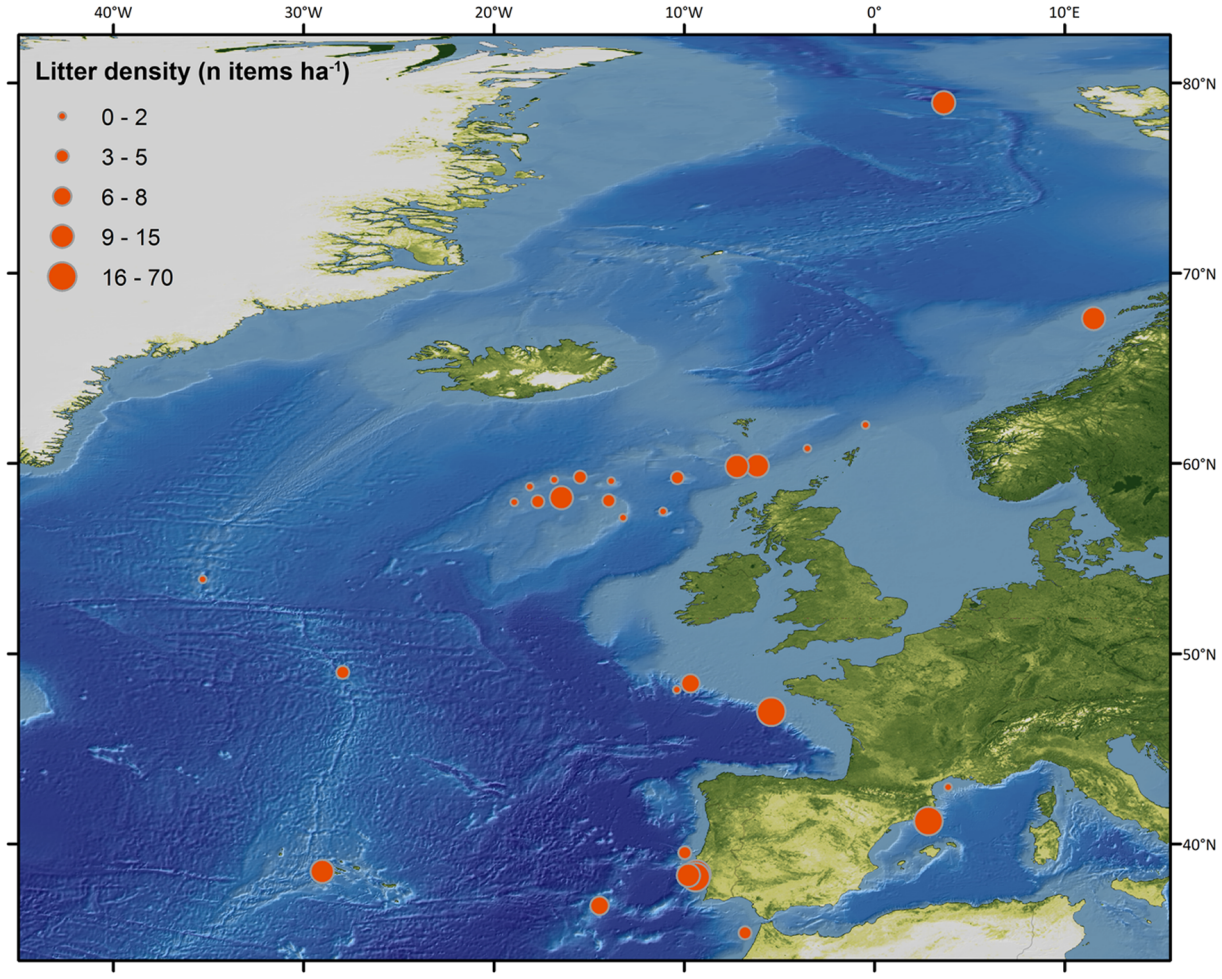
Litter densities (number of items ha^−1^) in different locations across European waters obtained with ROVs, towed camera systems, manned submersible and trawls.

**Figure 4:**
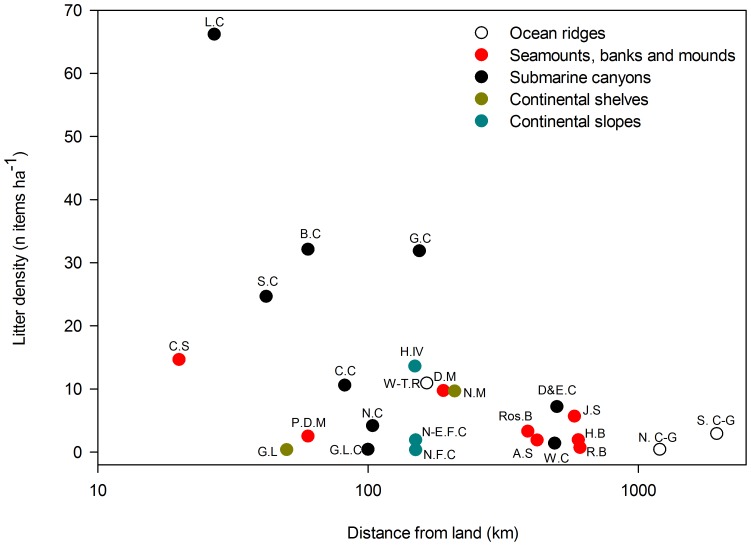
Litter densities (number of items ha^−1^) in different locations across European waters according to their closest distances from land. *x* axis is in a Log_10_ scale. A.S  =  Anton Dohrn Seamount, B.C  =  Blanes Canyon (NW Med.), C.C  =  Cascais Canyon, C.S  =  Condor Seamount, D&E.C  =  Dangeard & Explorer Canyons, D.M  =  Darwin Mounds, G.L.C  =  Gulf of Lion canyons (NW Med.), G.L  =  Gulf of Lion, G.C  =  Guilvinec Canyon, H.B  =  Hatton Bank, H.IV  =  HAUSGARTEN, station IV, J.S =  Josephine Seamount, L.C =  Lisbon Canyon, N.C  =  Nazaré Canyon, N.C-G  =  North Charlie Gibbs Fracture Zone, N-E.F.C  =  North-East Faroe-Shetland Channel, N.F.C  =  North Faroe-Shetland Channel, N.W =  Norwegian margin, P.D.M  =  Pen Duick Alpha/Beta Mound, R.B  =  Rockall Bank, Ros.B  =  Rosemary Bank, S.C  =  Setúbal Canyon, S.C-G  =  South Charlie Gibbs Fracture Zone, W.C  =  Whittard Canyon, W-T.R  =  Wyville-Thomson Ridge.

Litter was found at all sites and all depths (from 35 m down to 4500 m) sampled. Most common litter items included plastic bags, glass bottles and derelict fishing lines and nets (Figure 2). Locations with highest litter densities (>20 items ha^−1^) included the Lisbon Canyon, the Blanes Canyon, the Guilvinec Canyon, and the Setúbal Canyon (Table 1; Figure 3). Sites with intermediate litter density (between 10 and 20 items ha^−1^) were found on the Condor Seamount, the Wyville-Thomson Ridge, the continental slope of the HAUSGARTEN observatory and the Cascais Canyon (Figure 3). Low densities (between 2 and 10 items ha^−1^) were recorded on the Darwin Mounds, off the Norwegian margin, in Dangeard and Explorer Canyons, on the Josephine Seamount, in the Nazaré Canyon, on the Rosemary Bank, south of the Charlie-Gibbs Fracture Zone and on the Pen Duick Alpha and Beta Mounds (Figure 3). The lowest litter density (<2 items ha^−1^) was found on the Hatton Bank, the continental slope on the northern side of the Faroe-Shetland Channel, on the Anton Dohrn Seamount, in the Whittard Canyon, on the Rockall Bank, north of the Charlie-Gibbs Fracture Zone, and in the Gulf of Lion (in both the continental shelf and submarine canyons). Sites with higher litter density were found principally closer to shore (Figure 4), but there were exceptions, such as the samples from the Gulf of Lion where litter densities were low (Table 1).

**Figure 5:**
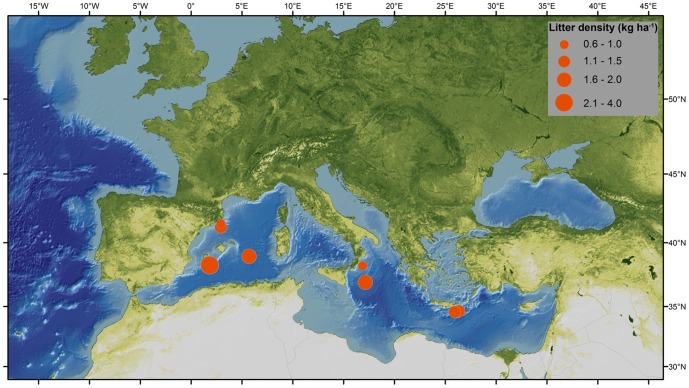
Litter densities (kg ha^−1^) in different locations across the Mediterranean Sea obtained from trawl surveys.

The sites sampled by trawling in the Mediterranean revealed a relatively even distribution of litter but with a higher density on the continental slope, south of Palma de Mallorca (western Mediterranean) with a mean (±SE) of 4.0±1.8 kg of litter ha^−1^ as opposed to densities ranging between 0.7 and 1.8 kg of litter ha^−1^ at the other sites (Figure 5).

**Figure 6:**
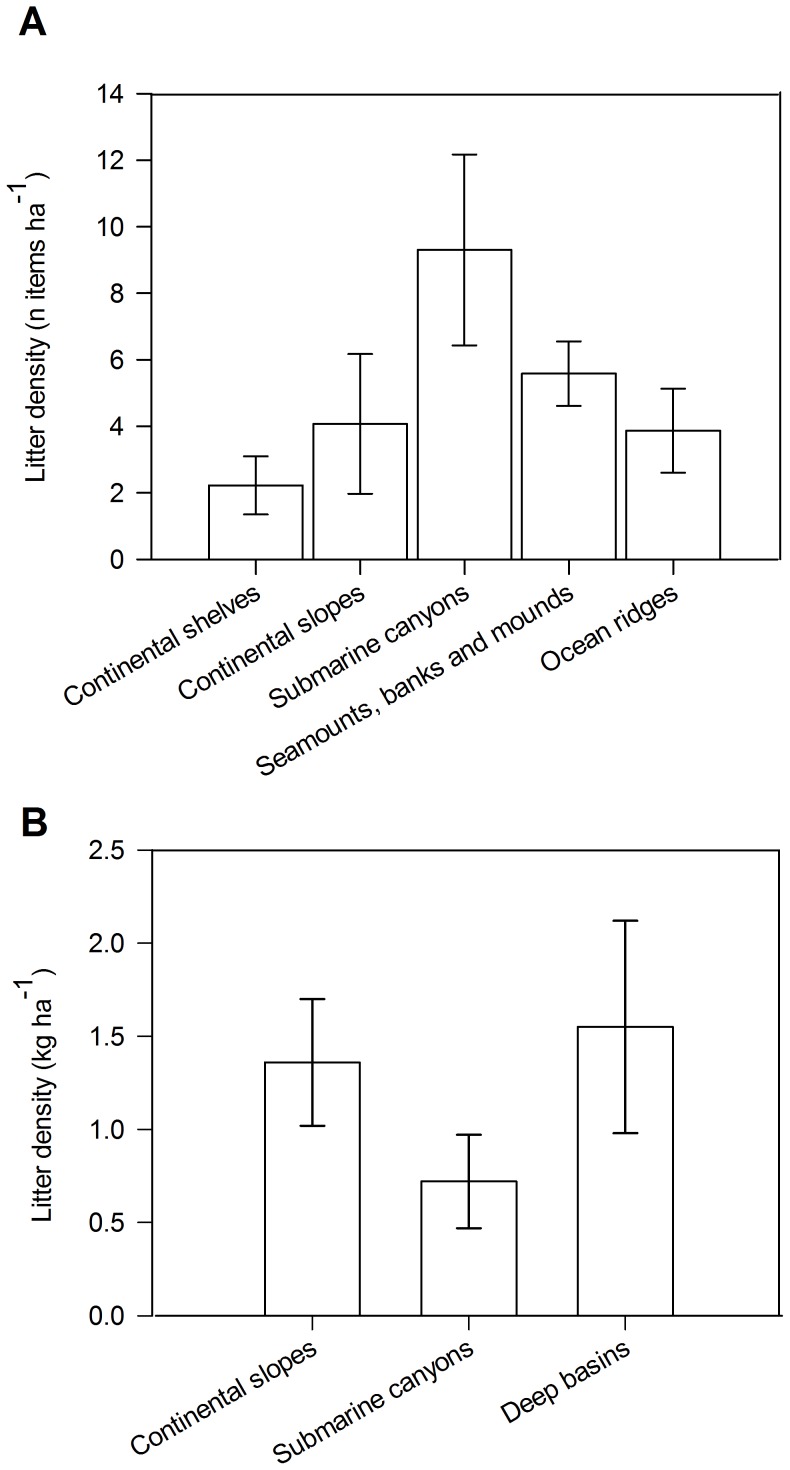
Mean litter density (± standard error) in A  =  number of items ha^−1^ and B  =  in kg of items ha^−1^, across different physiographic settings in European waters.

When grouping all sites into physiographic settings, there were significant differences in litter density (items ha^−1^) between the various groups (Kruskal-Wallis χ^2^ = 26.68; p<0.01; DF = 4). Multiple comparisons tests indicated that litter density in submarine canyons was significantly higher than those from all other physiographic settings, reaching an average (± SE) of 9.3±2.9 items ha^−1^ (Figure 6a). Litter density on seamounts, mounds and banks was similar to the densities found on the continental slopes with mean (± SE) densities of 5.6±1.0 and 4.1±2.1 items ha^−1^, respectively (Figure 6a). Mean (± SE) litter density for continental shelves and ocean ridges was 2.2±0.8 and 3.9±1.3 items ha^−1^, respectively (Figure 6a). For Mediterranean sites, where litter density was quantified by weight rather than number of items, no significant differences were found in litter density between the three different physiographic settings (Kruskal-Wallis χ^2^ = 3.88; p = 0.144; DF = 2). However, litter density in deep basins was slightly higher (1.55±0.57 kg ha^−1^) compared to continental slopes (1.36±0.34 kg ha^−1^) and submarine canyons (0.71±0.25 kg ha^−1^) (Figure 6b).

### Litter composition

There was a high variability in the composition of litter across the different sites (Table 3). A total of 546 litter items were encountered throughout all sites surveyed with imaging technology. Plastic and derelict fishing gear were the most abundant litter items. Plastic represented 41% of the litter items, whilst derelict fishing gear accounted for 34% of the total. Clinker, glass and metal were least common (1, 4 and 7%, respectively). Items classified as “other items” accounted for 13% of the litter items encountered in sites surveyed by imaging technology and included wood, paper/cardboard, clothing, pottery, and unidentified material. Analysis of litter density from trawl surveys found plastic to be the most common litter type to be recovered (found in 98% of the trawls), followed by clinker (73%), fabric (48%), derelict fishing gear (33%), metal (31%) and glass (28%).

**Table 3:** Composition of litter (%) in different locations on the seafloor of European waters.

Location	Derelict fishing gear	Glass	Metal	Plastic	Other items	Clinker
**ATLANTIC**						
***Continental slopes***						
North Faroe-Shetland Channel	100.0	0.0	0.0	0.0	0.0	0.0
North-East Faroe-Shetland Channel	100.0	0.0	0.0	0.0	0.0	0.0
***Continental shelf***						
Norwegian Margin	80.0	0.0	0.0	20.0	0.0	0.0
***Submarine canyons***						
Dangeard & Explorer Canyons	72.2	0.0	0.0	16.7	11.1	0.0
Nazaré Canyon	37.1	0.0	17.1	25.7	20.0	0.0
Lisbon Canyon	9.2	0.0	1.5	86.2	3.1	0.0
Setúbal Canyon	8.7	4.3	4.3	30.4	52.2	0.0
Cascais Canyon	9.1	0.0	0.0	54.5	36.4	0.0
Guilvinec Canyon	43.8	0.0	0.0	43.8	6.3	6.3
Whittard Canyon	28.6	7.1	14.3	42.9	0.0	7.1
***Seamounts, banks and mounds***						
Anton Dohrn Seamount	0.0	0.0	100.0	0.0	0.0	0.0
Condor Seamount	85.5	14.5	0.0	0.0	0.0	0.0
Josephine Seamount	42.9	28.6	14.3	0.0	14.3	0.0
Hatton Bank	87.5	0.0	12.5	0.0	0.0	0.0
Rockall Bank	33.3	0.0	66.7	0.0	0.0	0.0
Rosemary Bank	66.7	0.0	33.3	0.0	0.0	0.0
Pen Duick Alpha/Beta Mound	75.0	0.0	25.0	0.0	0.0	0.0
Darwin Mounds	10.0	0.0	15.0	60.0	15.0	0.0
***Ocean ridges***						
North Charlie Gibbs Fracture Zone	0.0	0.0	100.0	0.0	0.0	0.0
South Charlie Gibbs Fracture Zone	0.0	28.6	28.6	28.6	14.3	0.0
Wyville-Thomson Ridge	85.7	0.0	14.3	0.0	0.0	0.0
**MEDITERANEAN**						
***Continental slopes***						
Calabrian Slope (Central Med.)	13.2	0.0	8.4	36.2	26.6	15.5
Western Mediterranean Slope	21.6	0.6	0.2	12.1	0.6	64.9
Crete-Rhodes Ridge (E. Med.)	1.6	9.3	6.0	17.0	20.5	45.5
Blanes slope (NW Med.)	2.3	7.9	8.4	12.6	11.6	57.1
***Continental shelf***						
Gulf of Lion (NW Med.)	0.0	0.0	0.0	88.9	11.1	0.0
***Submarine canyons***						
Blanes Canyon (NW Med.)	3 (0.2)	3 (4.9)	6 (2.2)	78 (76.3)	9 (1.7)	0 (14.7)
Gulf of Lion Canyons (NW Med.)	0.0	0.0	0.0	67.3	32.7	0.0
***Deep basins***						
Algero-Balearic Basin (W. Med.)	16.5	0.8	29.6	14.0	2.1	37.0
Crete-Rhodes Ridge (E. Med.)	0.0	9.7	25.0	19.5	7.2	38.5
Calabrian Basin (Central Med.)	0.5	6.7	0.7	5.9	36.1	50.1
**ARCTIC**						
***Continental slope***						
HAUSGARTEN, station IV	2.5	2.5	2.5	60	32.5	0

*Numbers in parentheses refer to trawl surveys.

**Figure 7:**
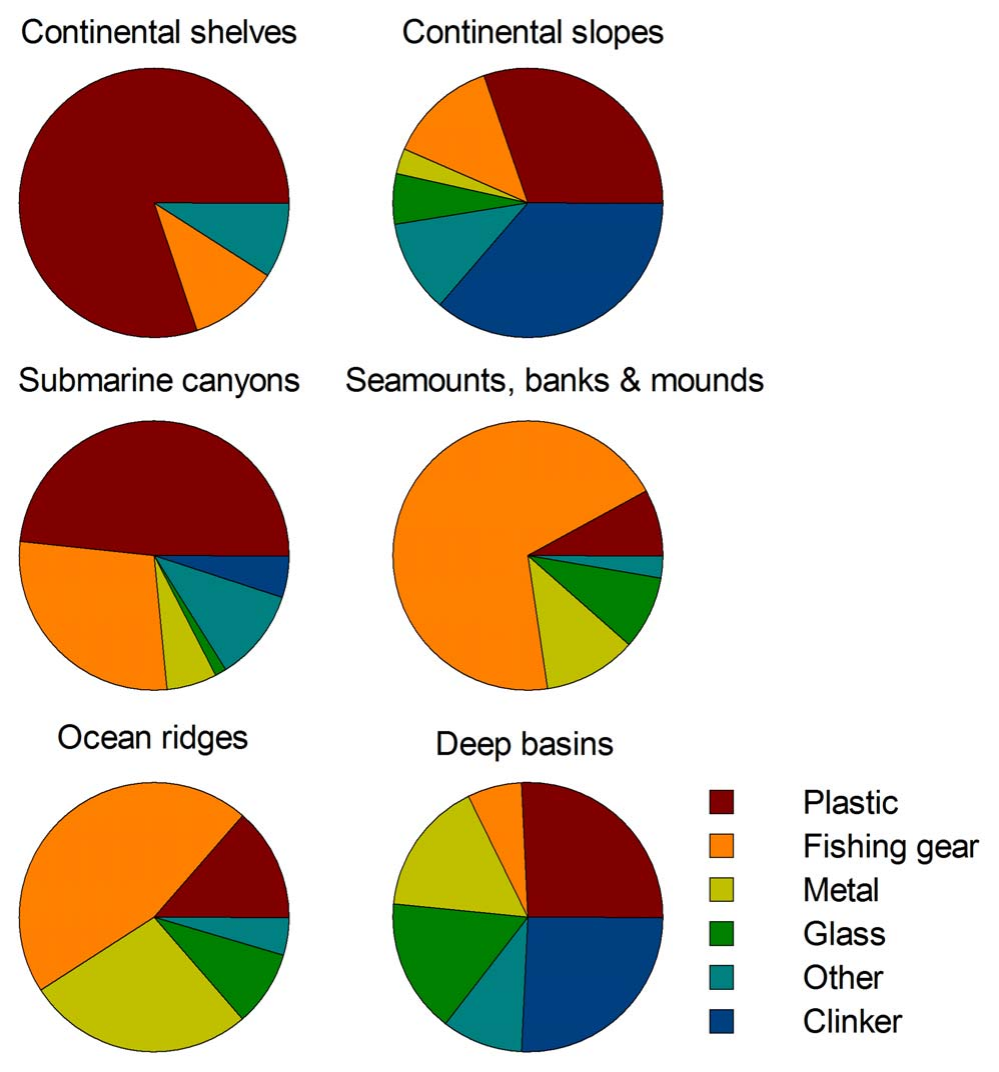
Litter composition in different physiographic settings across European waters.

Results from ANOSIM showed that there were significant differences in litter composition between physiographic settings (1-way ANOSIM; Global R = 0.32; p<0.001), the analysis also showed some settings to be similar (Table S1). There were no significant differences between litter composition in submarine canyons and continental shelves (R = 0.01; p = 0.58). According to SIMPER analysis (Table S2), the similarity in composition between submarine canyons and continental shelves was mostly driven by plastic. Plastic was the dominant litter category for both settings (Figure 7). Litter composition on ocean ridges and on seamounts, banks and mounds did not show significant differences in litter composition (R = 0.17; p = 0.06), due to a predominance of derelict fishing gear (Figure 7). Finally, litter composition found on continental slopes was similar to deep basins (R = −0.11; p = 0.87). Clinker and plastic were the categories contributing most to the similarities between these two physiographic settings.

## Discussion

The occurrence of litter on the seafloor has been far less investigated than in surface waters or on beaches, principally because of the high cost and the technical difficulties involved in sampling the seafloor at bathyal and abyssal depths [21], [60]. Considering such limitations and poor knowledge on litter accumulation in deep waters, every survey is of great value for obtaining information on litter density and distribution. In the present study, we integrated data collected during numerous cruises over a large regional scale into a single analysis, providing insight on the density and composition of litter across a wide variety of seafloor settings and over a large geographical area in European waters. Although standardisation of the data permitted comparisons between sites, dissimilarities in the sampling equipment implies that the results should be treated with caution. Furthermore, differences in the areas of the seafloor surveyed between locations may lead to overestimations or underestimations of the litter density. Also, studying litter from trawls introduces the issue of quantification units (number vs. weight), with no correct solution. When using number of items, certain litter categories may be overestimated such as plastic or glass that can break into many small pieces. As a counterpart, if weight is used, the abundance of litter type with different weights (e.g. heavy clinker vs. light plastic) cannot be compared. Ideally, both units for litter quantification will help to understand better trends, but the EU Marine Strategy Framework Directive stresses that for monitoring litter in the marine environment, number is mandatory whilst weight is only recommended [2].

Litter was found at all the locations surveyed, from sites close to population centres such as the Gulf of Lion or the Lisbon Canyon to as far as the South Charlie-Gibbs Fracture Zone on the Mid-Atlantic Ridge, located at about 2000 km from land. Litter was found from shallow waters (35 meters in Gulf of Lion) down to 4500 meters (Cascais Canyon). Such records were not surprising, as litter is known to be present in all seas and oceans of the planet, as remote as the Southern Ocean [21] and at depths as deep as 7216 m in the Ryuku trench, south of Japan [61]. The range of litter densities found on our study sites was within the same order of magnitude to the ones found on the seabed in other parts of the globe (North America [55], [62], [63], China [54], Japan [64], [65]) and for other locations in Europe [28], [44], [45], [47], [48]. On the other hand, macro litter densities on the seabed were higher than reported for surface waters [32], [66]–[69]. At the surface, floating litter tends to accumulate in frontal areas but eventually reaches the seabed when heavily covered by fouling organisms [70] or loaded with sediments. Contrary to a common notion that most plastic items float at the sea surface it has been estimated that 70% of the plastic sinks to the seafloor [23]. This results in macro litter accumulation on the seabed rather than in the open sea [21]. For example, on the seafloor of the Mediterranean Sea, our data showed much higher litter densities (0.4 to 48 litter items ha^−1^) than that estimated to float at the surface (0.021 items ha^−1^; [1]). Alternatively, floating litter may be transported for considerable distances and get washed ashore [71], [72]. Litter density on the coastline is typically higher than on the seafloor given that there is an additional input of waste coming from inland sources (e.g. man-made drainage systems, recreational usage, rivers, winds, etc.) [71], [73]. On European coasts, litter densities can exceed 30,000 litter items per linear km [1], [41], [74], while much higher densities have been reported for beaches in Indonesia [75] or on the beaches along Armaçao dos Buzios, Rio de Janeiro, Brazil [76]. However, comparisons between studies are challenging considering differences in the size of the litter items sampled and the sampling methodology used [77].

Our data showed a general increase in litter density in locations closer to the shore, a pattern previously reported for the French Mediterranean coast [47] and off California [55]. Nevertheless, low litter densities in some near-shore sites (e.g. Gulf of Lion or Faroe-Shetland channel) suggest that many other factors (such as geomorphology, hydrography and human activity) affect litter distribution and accumulation rates [29]. In the Gulf of Lion, Galgani et al. [47] suggested that low litter density on the shelf was caused by strong water flow from the Rhone River, transporting litter down south to deeper waters. A similar situation occurs in Monterey Bay where sediment and litter are being swept off the continental shelf down into Monterey Canyon [78]. Such phenomena may explain why continental shelves were the settings with overall lowest litter density, whilst submarine canyons had the highest litter concentration. Litter levels on seamounts, banks, mounds and ocean ridges were characterised by intermediate levels when compared to other physiographic settings. They are typically located far away from coastal areas where the main anthropogenic activities include fishing [79] and seabed mining [80], [81]. The presence of litter on these settings is of concern because they harbor Vulnerable Marine Ecosystems (VMEs) (such as cold-water corals and hydrothermal vents) that have reduced capacity to recover from disturbance events and for which conservation is a global priority [82].

The types of accumulated litter can provide an indication on the human activities impacting a particular location. However, one must be cautious and consider the differences in the buoyancy and longevity of the different types of litter. For example, while some plastics sink to the seafloor, others float on the surface and are able to travel great distances before eventually sinking far from their initial dumping locations, following biofouling and degradation [23]. On the other hand, glass, metal and clinker will sink rapidly and are expected to be recovered from the seafloor close to sites where they were initially released. Cardboard and fabrics (of organic origin) will break down quickly, implying that such items will not reach the deep ocean with the frequency of more resistant materials such as plastic and negatively buoyant items such as glass, metal and clinker. Although it is difficult to determine the exact source of the litter observed on the seafloor, the dominant litter category can be used as an indicator to separate ocean and terrestrial sources [15], [29], [31], [78]. Plastic (other than derelict fishing gear) was the most abundant litter category in submarine canyons, continental shelves and continental slopes. The predominance of plastics in submarine canyons reaffirms that litter accumulation in these habitats comes from coastal and land sources and that submarine canyons act as conduits for litter transport from continental shelves into deeper waters [21], [28], [29], [31], [47], [78]. Therefore, submarine canyons can be considered to be accumulation zones of land-based marine litter in the deep sea. In fact, submarine canyons are areas where macrophyte detritus that originates from coastal areas accumulates in high quantities. This results in a localised increase of organic matter and high abundances of associated fauna, dominated by deposit and suspension-feeding invertebrates [83]–[85]. Since some deposit-feeders (e.g. holothurians) have been shown to select plastic fragments over sediment grains under laboratory conditions [7], the accumulation of plastics in submarine canyons could have detrimental effects for these ecologically important deep-sea organisms. Furthermore, plastic fragments contain a wide variety of persistent organic pollutants (POPs) that may accumulate in the consumer's tissues and can be transferred upwards in the trophic webs to predators, including humans [86].

Derelict fishing gear was the main litter item found on seamounts, banks, mounds and ocean ridges implying that, unlike submarine canyons, fishing activities are the major source of litter at those settings. Seamounts and banks are targeted by commercial fishing activities as they are often highly productive areas supporting dense aggregations of commercially valuable fish and shellfish [87]. At other locations where recreational [55], [88] and commercial [28], [54], [62], [89] fishing activities are intense, derelict fishing gear dominated the litter on the seabed. It was beyond the scope of this study to evaluate the impacts caused by derelict fishing gear, but numerous studies have shown diverse impacts including ghost fishing [16], [90] and entanglement by sessile invertebrates such as corals [15], as well as causing damage to fishing equipment [91]. Discarded trawl gear can also have a compounding effect by trapping more mobile litter resulting in a litter ‘depot’ that has a greater impact than single pieces of litter [31]. Since most fishing equipment (lines and nets) is made mostly of highly resistant plastics, such negative effects will likely persist for a long time. Sites located in deep basins and continental slopes were dominated by clinker. Clinker, the residue of burnt coal, was commonly dumped from steam ships from the late 18^th^ century and well into the 20^th^ century. In the Mediterranean Sea, its occurrence on the deep seafloor has been shown to coincide with such shipping routes [29]. However, it is important to acknowledge that in this study, deep basins and continental slopes were principally sampled by trawling and it is difficult to determine if the differences in litter composition with other physiographic settings are the results of differences in the sampling methodology, particularly since clinker is difficult to identify from underwater footage. Indeed, clinker was present in non-quantitative trawls undertaken at HAUSGARTEN (Bergmann, unpublished data), but could not be detected on images from the seafloor. Similarly, a high abundance of clinker was recovered from trawl surveys in Blanes Canyon that could not be identified in analysis of ROV footage from the same area (Table 3). Given that most of the clinker present on the seafloor was dumped over 100 years ago, sedimentation will have buried it, which would explain the differences in clinker quantification between images and trawl data. The deep seafloor is a passive accumulation area for litter, integrating information over long-time periods. If trawls are able to recover heavy clinker deposited on the seafloor over a century ago, these gears must be retrieving at the same time all of the lighter and most recent litter items, such as plastic for example, that have been accumulating only in the last 50 years. Overall, the composition of litter found on the seafloor showed some dissimilarity with the composition found on the coasts or in surface waters. Although plastics are dominant in all settings [70], some areas of the seafloor investigated here and elsewhere [28], [44], [45], [54], [78] harbour significant quantities of non-buoyant litter such as glass, metal and clinker, directly dumped from ships but that are seldom found in surface waters [41], [68] or on the coasts [41], [72]. The coasts and surface waters are a source of litter items for the open seas and all this litter, sooner or later, will sink to the seafloor where it accumulates.

The most common method used to provide data on benthic marine litter has been trawling, typically as a parallel objective to surveys directed to fish or benthic organism sampling [53]. With the recent development of optical methods fitted to platforms such as submersibles, ROV and drop-down systems, the use of underwater imaging technology has greatly increased our ability to quantify deep-sea litter. Both methods (imaging technology and trawling) have distinct assets for studying benthic litter that should be used in conjunction to best understand the dynamics of pollution on the seafloor. Video surveys can provide data for areas where topography is complex (e.g seamounts or canyon walls), habitats made by structure-building organisms (e.g. cold-water corals), or dynamic systems (e.g. hydrothermal vents and cold seeps), that cannot be accessed with a trawl [53]. Furthermore, imaging is a non-intrusive method that does not remove benthic organisms or damage the environment. On the other hand, a trawl has the advantages of recovering litter items of very small size (e.g. small plastic fragments) or that are buried in the sediments (e.g. clinker), which otherwise would not be detected through imaging technology. In addition, litter items collected with a trawl can be analysed in the laboratory to obtain further important information, such as state of degradation or colonisation by fouling organisms [92]. Such data will help understand sinking processes of plastic, facilitate the identification of their location of arrival into the ocean and provide information on the impacts of litter on marine organisms.

The large quantities of litter reaching the deep ocean floor is a major issue worldwide, yet little is known about its sources, patterns of distribution, abundance and, particularly, impacts on the habitats and associated fauna [1]. At present, density of litter in the deep sea is lower than found on some heavily polluted beaches [33], [93], but unlike the coastal zone, only a tiny fraction of the (deep) seafloor has been surveyed to date. Furthermore, microplastic accumulation may become an important component of pollution in deep-sea ecosystems [94] that urgently needs to be evaluated. Our results for European waters show that litter sources are distinct across different physiographic settings and that their abundance is variable, most probably guided by a complex set of interactions between physiography, anthropogenic activities and hydrography. It is important that in the future, large-scale assessments are done in a standardised manner to understand fully the scale of the problem and set the necessary actions to prevent the accumulation of litter in the marine environment.

## Supporting Information

Table S1 Results of analyses of similarity (ANOSIM) evaluating variation in the composition of litter among physiographic settings. RIDGE: ocean ridges; CANY: submarine canyons; SHELF: continental shelves; SLOPE: continental slopes; SBM: seamounts, banks and mounds; BASIN: deep basins.
(DOCX)

Table S2 Similarity percentage analysis (SIMPER) of litter composition for each pooled physiographic settings (based on similarities revealed by ANOSIM) and the contribution of litter category to group similarity.
(DOCX)
