# Supplementary material for: Marine Litter Distribution and Density in European Seas, from the Shelves to Deep Basins
Source: PLoS One. 2014 Apr 30;9(4):e95839. doi: 10.1371/journal.pone.0095839 (PMC4005782; doi:10.1371/journal.pone.0095839)
Supplement: Table S1 — Results of analyses of similarity (ANOSIM) evaluating variation in the composition of litter among physiographic settings. RIDGE: ocean ridges; CANY: submarine canyons; SHELF: continental shelves; SLOPE: continental slopes; SBM: seamounts, banks and mounds; BASIN: deep basins. (DOCX) [file pone.0095839.s001.docx]

**Table S1.** Results of analyses of similarity (ANOSIM) evaluating variation in the composition of litter among physiographic settings. RIDGE: ocean ridges; CANY: submarine canyons; SHELF: continental shelves; SLOPE: continental slopes; SBM: seamounts, banks and mounds; BASIN: deep basins

| **Comparison** | **R** | **p-value** |
| --- | --- | --- |
| RIDGE vs. CANY | 0.213 | 0.001 |
| RIDGE vs. SBM | 0.165 | 0.058 |
| RIDGE vs. SLOPE | 0.603 | 0.001 |
| RIDGE vs. SHELF | 0.570 | 0.001 |
| RIDGE vs. BASIN | 0.350 | 0.006 |
| CANY vs. SBM | 0.236 | 0.001 |
| CANY vs. SLOPE | 0.243 | 0.001 |
| CANY vs. SHELF | 0.011 | 0.581 |
| CANY vs. BASIN | 0.191 | 0.027 |
| SBM vs. SLOPE | 0.590 | 0.001 |
| SBM vs. SHELF | 0.547 | 0.001 |
| SBM vs. BASIN | 0.517 | 0.003 |
| SLOPE vs. SHELF | 0.224 | 0.001 |
| SLOPE vs. BASIN | 0.103 | 0.867 |
| SHELF vs. BASIN | 0.633 | 0.001 |
