# Supplementary material for: Marine Litter Distribution and Density in European Seas, from the Shelves to Deep Basins
Source: PLoS One. 2014 Apr 30;9(4):e95839. doi: 10.1371/journal.pone.0095839 (PMC4005782; doi:10.1371/journal.pone.0095839)
Supplement: Table S2 — Similarity percentage analysis (SIMPER) of litter composition for each pooled physiographic settings (based on similarities revealed by ANOSIM) and the contribution of litter category to group similarity. (DOCX) [file pone.0095839.s002.docx]

**Table S2.** Similarity percentage analysis (SIMPER) of litter composition for each pooled physiographic settings (based on similarities revealed by ANOSIM ) and the contribution of litter category to group similarity.

| **Pooled physiographic settings** | **Average group similarity:** | **Litter category** | **Average Similarity** | **Ratio**  **(Similarity/Standard deviation)** | **Percentage Contribution** |
| --- | --- | --- | --- | --- | --- |
|  |  |  |  |  |  |
|  |  |  |  |  |  |
| RIDGE and SBM | 48.82 | Fishing gear | 44.08 | 1.03 | 90.29 |
|  |  |  |  |  |  |
| CANY and SHELF | 47.91 | Plastic | 39.17 | 1.04 | 81.75 |
|  |  |  |  |  |  |
| BASIN and SLOPE | 48.95 | Plastic | 22.49 | 1.45 | 45.72 |
|  |  | Clinker | 17.13 | 0.86 | 34.82 |
|  |  | Fishing gear | 2.63 | 0.27 | 5.35 |
|  |  | Other | 4.31 | 0.46 | 8.77 |
|  |  |  |  |  |  |
